# Cost-effectiveness of antenatal corticosteroids and tocolytic agents in the management of preterm birth: A systematic review

**DOI:** 10.1016/j.eclinm.2022.101496

**Published:** 2022-06-03

**Authors:** Elizabeth Sebastian, Chloe Bykersma, Alexander Eggleston, Katherine E. Eddy, Sher Ting Chim, Rana Islamiah Zahroh, Nick Scott, Doris Chou, Olufemi T. Oladapo, Joshua P. Vogel

**Affiliations:** a Maternal, Child and Adolescent Health Program, Burnet Institute, Melbourne, Australia; b Faculty of Medicine, Nursing and Health Sciences, Monash University, Melbourne, Australia; c Gender and Women's Health Unit, Centre for Health Equity, Melbourne School of Population and Global Health, University of Melbourne, Melbourne, Australia; d UNDP/UNFPA/UNICEF/WHO/World Bank Special Programme of Research, Development and Research Training in Human Reproduction (HRP), Department of Sexual and Reproductive Health and Research, World Health Organization, Geneva, Switzerland; e School of Public Health and Preventive Medicine, Monash University, Melbourne, Australia

**Keywords:** Cost-effectiveness, Economic evaluation, Preterm birth, Antenatal corticosteroids, Tocolysis, Tocolytic

## Abstract

**Background:**

Preterm birth is a leading cause of neonatal mortality and morbidity, and imposes high health and societal costs. Antenatal corticosteroids (ACS) to accelerate fetal lung maturation are commonly used in conjunction with tocolytics for arresting preterm labour in women at risk of imminent preterm birth.

**Methods:**

We conducted a systematic review on the cost-effectiveness of ACS and/or tocolytics as part of preterm birth management. We systematically searched MEDLINE and Embase (December 2021), as well as a maternal health economic evidence repository collated from NHS Economic Evaluation Database, EconLit, PubMed, Embase, CINAHL and PsycInfo, with no date cutoff. Eligible studies were economic evaluations of ACS and/or tocolytics for preterm birth. Two reviewers independently screened citations, extracted data on cost-effectiveness and assessed study quality using the Consolidated Health Economic Evaluation Reporting Standards (CHEERS) statement.

**Findings:**

35 studies were included: 11 studies on ACS, eight on tocolytics to facilitate ACS administration, 12 on acute and maintenance tocolysis, and four studies on a combination of ACS and tocolytics. ACS was cost-effective prior to 34 weeks’ gestation, but economic evidence on ACS use at 34-<37 weeks was conflicting. No single tocolytic was identified as the most cost-effective. Studies disagreed on whether ACS and tocolytic in combination were cost-saving when compared to no intervention.

**Interpretation:**

ACS use prior to 34 weeks’ gestation appears cost-effective. Further studies are required to identify what (if any) tocolytic option is most cost-effective for facilitating ACS administration, and the economic consequences of ACS use in the late preterm period.

**Funding:**

UNFPA/UNICEF/WHO/World Bank Special Programme of Research, Development and Research Training in Human Reproduction (HRP), a cosponsored programme executed by WHO.

**Research in context**

**Evidence before this study**

Efficacy evidence indicates that antenatal corticosteroids (ACS) prior to 34 weeks’ gestation for women at risk of imminent preterm birth significantly reduces neonatal morbidity and mortality. Though there is relatively less evidence on effects of ACS in the late preterm period (34 to <37 weeks’ gestation), they might reduce neonatal respiratory morbidity but could also increase neonatal hypoglycaemia. Multiple drug classes have been evaluated for tocolysis in women with spontaneous preterm labour. Some tocolytic drugs can effectively prolong pregnancy – providing time for ACS administration and/or transfer to higher level care – but tocolytic drugs have not yet been shown to independently improve substantive perinatal health outcomes. We identified a 2009 health technology assessment that broadly evaluated the economic effects of test-treatment interventions in preterm labour, however the cost-effectiveness of ACS and/or tocolytics only were not specifically reported.

**Added value of this study**

We searched MEDLINE, Embase and a repository of maternal health economic evaluations derived from six economic and health databases. Available economic studies of ACS and/or tocolytics were largely conducted in high-income countries. ACS prior to 34 weeks’ gestation appears cost-effective, though economic evidence from the USA on ACS use in late preterm birth indicates that its cost-effectiveness varies depending on which health outcomes are considered. Some studies suggest that tocolysis to facilitate ACS administration was not cost-saving, but may be cost-effective. No single tocolytic option was identified as dominant in the management of spontaneous preterm labour.

**Implications of all the available evidence**

ACS prior to 34 weeks’ gestation is cost-effective in high-income countries. There is limited economic evidence from low-to-middle-income countries, though modelling suggests ACS implementation and scale up would likely be cost-effective in these contexts. In light of the limited and conflicting evidence on tocolytics for spontaneous preterm labour, it is not possible to conclude what (if any) tocolytic option is the most cost-effective. Further, robust economic evaluations on ACS at 34-<37 weeks’ gestation, tocolytics alone, and ACS and tocolytics in combination are required, particularly those that explore cost-effectiveness in resource-limited settings.

Alt-text: Unlabelled box

## Introduction

An estimated 14.84 million infants are born preterm worldwide every year.^1^ Complications relating to preterm birth are the leading cause of mortality in children under 5 worldwide.^2^ Neonatal complications of preterm birth can include respiratory distress syndrome (RDS), bronchopulmonary dysplasia (BPD), necrotising enterocolitis (NEC), intraventricular haemorrhage (IVH), and several other serious morbidities.^3^ Over the longer term, babies born preterm have higher rates of neurodevelopmental disabilities, as well as more frequent hospitalisations, incurring large societal costs.^3^ In Australia, an estimated A$1.4 billion is spent annually on healthcare and educational costs associated with preterm children until 18 years of age.^4^

In 2015 WHO released evidence-based guidelines on the use of interventions to improve preterm birth outcomes.^5^ These interventions include the use of antenatal corticosteroids (ACS) and tocolytics, as well as several interventions used in the care of preterm infants. ACS (typically intramuscular dexamethasone or betamethasone) can cross the placenta and accelerate fetal lung maturation.^6^ When administered to women at risk of imminent preterm birth prior to 34 weeks’ gestation, ACS can prevent perinatal and neonatal death, RDS and IVH, without causing maternal or newborn harms.^7^ WHO thus recommended that ACS can be used for this indication, provided that a minimum standard of maternal and preterm newborn care is available.^5^ While WHO does not recommend the routine use of tocolytics for women in preterm labour (in light of the lack of substantive effects on perinatal health outcomes), the guideline panel acknowledged that some tocolytic options prolong pregnancy by 2-7 days, providing a window for ACS administration or in-utero transfer to a higher-level care facility.^8^ In such instances, nifedipine is the preferred tocolytic drug.^5^ Acute tocolysis is recommended in several high-income countries,^9^ and observational evidence indicates that some tocolytics (such as betamimetics and calcium channel blockers) are used for preterm labour management in low- and middle-income countries (LMICs).^10^

Evaluating healthcare interventions from both health and economic perspectives allows policymakers, clinicians and other stakeholders to identify the most efficient (or cost-effective) healthcare strategies to maximise health benefits at a population level.^11^ In resource-limited settings, cost is often a key consideration in the decision to implement interventions at scale. While several economic evaluations have been conducted on ACS and tocolytics in preterm birth, to date no review has synthesized all available economic evidence. Cochrane systematic reviews on the effectiveness of ACS and different tocolytic options for preterm birth did not pre-specify outcomes related to cost or cost-effectiveness.^7^^,^^12^, ^13^, ^14^, ^15^, ^16^, ^17^, ^18^, ^19^ In this study, we aimed to synthesize all available evidence on the cost-effectiveness of ACS and tocolytics as individual or co-interventions for improving preterm birth outcomes.

## Methods

This review is reported according to the Preferred Reporting Items for Systematic reviews and Meta-Analyses (PRISMA) 2020 checklist.^20^ As a systematic review of published studies, ethical approval was not required nor sought. The scoping review protocol is registered at DOI: 10.17605/OSF.IO/JWTGE.

### Eligibility criteria

Eligible studies were those that assessed the cost-effectiveness of ACS and/or tocolytic therapy for preterm birth. The primary outcome of interest was the incremental cost effectiveness ratio (ICER) (i.e., the change in cost and effectiveness when an intervention is compared to alternative intervention) of these two interventions, whether compared to no treatment or alternative treatment. We also extracted any available data on other relevant health economic measures, such as estimates of quality-adjusted life-years (QALYs) (years of life lived with perfect health), cost, cost savings, or cost benefit.

### Information sources, search strategy and selection process

Our research team has previously conducted a broad scoping review to identify economic evaluations of any maternal health intervention.^21^ In brief, eligible studies were sought from specialist health economic databases (NHS Economic Evaluation Database and EconLit) and medical databases (PubMed, Embase, CINAHL, and PsycInfo) using a structured search conducted on 20 November 2020. Eligible studies for that scoping review were full economic evaluations that assessed cost-benefit, cost-effectiveness, and/or cost-utility for women at any stage of pregnancy, childbirth, and up to six weeks postpartum. Studies of any intervention directed primarily towards improving maternal health outcomes were eligible, though interventions related to pre-conception care, ectopic pregnancy, early pregnancy loss, or management of abortion were not included. The scoping review had no restrictions in terms of comparator, publication date, country, or language. For the current review of cost-effectiveness studies of ACS or tocolytic therapy, we searched all 923 studies included in the scoping review database using synonyms of ‘antenatal corticosteroid’ and ‘tocolytic’, as well as reviewing any study conducted in women experiencing preterm birth (Appendix S1).

In order to update the search with more recent studies and capture studies not indexed by NHS EED, we searched MEDLINE and Embase for relevant studies with no setting or language restrictions on 14 December 2021. The search strategy was designed with assistance from an information specialist, using search terms related to ‘antenatal corticosteroid’, ‘tocolytic’, ‘preterm birth’ and ‘economic evaluation’ (Appendix S1).

For both searches at least two review authors independently screened all titles and abstracts, assessed full texts of potentially eligible studies, and extracted data (disagreements were resolved by discussion). Covidence software was used for title and abstract and full text screening. Studies were included if the intervention was directly related to use of an ACS and/or tocolytic, regardless of drug type. Studies related to progestational agents were not included as they pertained to prevention - rather than management - of preterm labour. In addition, reference lists of each of the included studies were reviewed to identify any additional eligible studies.

### Data extraction, synthesis, and quality assessment

Data were extracted by two authors independently using a pre-designed Excel spreadsheet adapted from a 2021 systematic review of cost-effectiveness studies by Aziz et al.^22^ Extracted data were primarily descriptive, including: country, setting, funding, study design, economic evaluation type, analytic perspective, currency, year of costs, time horizon, and data sources used. Available cost data and incremental cost-effectiveness ratios (ICER) were extracted for each study. Any disagreements on data extraction were resolved through discussion or consultation with a third author. Costs were reported as described in an included study and were not converted to a single currency or year of costs. Results were summarised in tables and reported narratively. Methodological quality of included studies was assessed using the International Society of Pharmacoeconomics and Outcomes Research (ISPOR) Taskforce Consolidated Health Economic Evaluation Reporting Standards (CHEERS) statement,^23^ as recommended by Wijnen et al.^24^ Three quality categories were adopted for the CHEERS score (a maximum score of 24) – high (>75%), moderate (50-74%), and low (<50%) as used by Zakiyah et al.^25^ Two authors independently assessed the quality of each study using this framework, with disagreements resolved through discussion or consulting a third author.

This work was financially supported by UNDP/UNFPA/UNICEF/WHO/World Bank Special Programme of Research, Development and Research Training in Human Reproduction (HRP), a cosponsored programme of the World Health Organization.

### Role of funding

The funder organization had no direct role in the study design, data collection, analysis, or interpretation. Two staff members of HRP/WHO were co-authors, and provided input to the study design, analysis and findings.

## Results

### Characteristics of included studies

**Figure 1:**
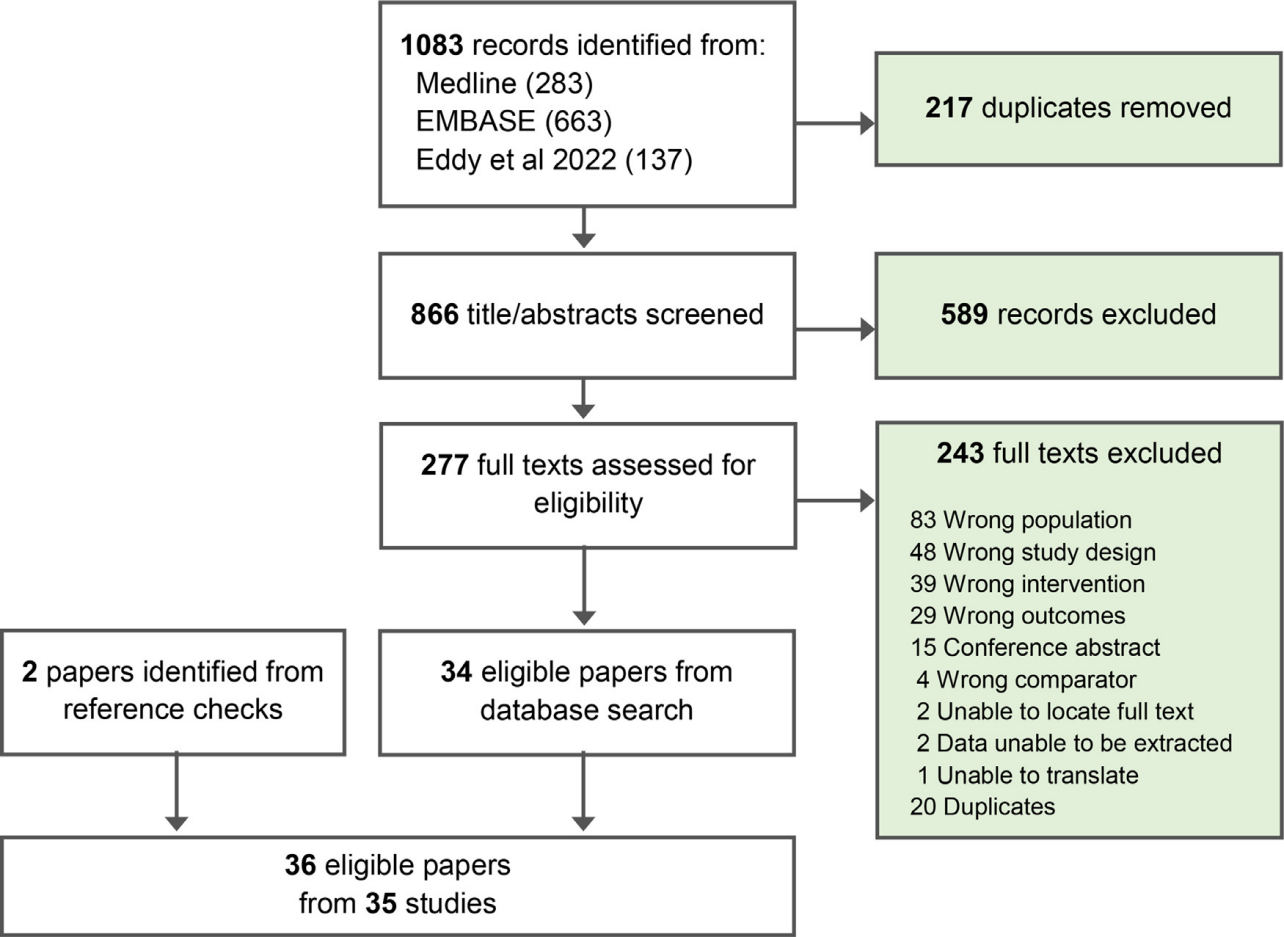
PRISMA flow diagram.

The combined searches identified 1083 citations, of which 34 were eligible (Figure 1). Two further studies were identified from reference list review of the included studies. A total of 35 studies from 36 citations were included in this review. One study was an abstract only and the full text could not be recovered. Among included studies, 11 pertained only to use of ACS (Table 1), 20 to use of tocolytics (Table 2), and four involved a combination of ACS and tocolytics (Table 3).

**Table 1:** Characteristics of included studies assessing cost-effectiveness of antenatal corticosteroids for preterm birth.

Study	Country	Care setting	Intervention	Study population	Aim	Design / analytic approach	Year of cost estimates	Type of evaluations (main outcomes)	Analytic viewpoint (perspective)	Time horizon (for effects)	CHEERS overall quality assessment
Antenatal corticosteroids prior to 34 weeks’ gestation											
Egberts 1992^29^	Netherlands	Inpatient facility	ACS (not specified)	< 30 weeks	Calculate the costs of various types of treatment to prevent or alleviate RDS in preterm neonates using data from a well-defined population of preterm infants.	Costs alongside retrospective cohort study	1990	Costs; cases of respiratory distress syndrome, mortality, cost per extra survivor	Not specified	Period of hospitalization until neonatal discharge	Moderate (16/22)
Morales 1986^30^	USA	Tertiary hospital	ACS (dexamethasone)	28 to 33 weeks	To establish whether the antenatal administration of corticosteroids results in improved neonatal outcome in gestations with premature rupture of membranes and to determine whether there is in- creased risk of neonatal and maternal infection.	Costs alongside randomised control trial	Not specified	Costs; neonatal morbidity^a^, neonatal hospitalization (days), maternal infection	Not specified	Period of hospitalisation until neonatal discharge	Low (10.5/23)
Mugford 1991^31^	UK	Tertiary hospital	ACS (not specified)	<35 weeks	To present estimates of the likely effects of giving corticosteroids to women expected to deliver preterm, and giving surfactant to babies at high risk of developing hyaline membrane disease, on health service costs.	Decision tree model	1989	Costs; respiratory distress syndrome; survival	Health service	Period of hospitalisation until neonatal discharge	Moderate (15.5/22)
Ogata 2016^26^	Brazil	University hospital	ACS (betamethasone or dexamethasone)	26-27 weeks, 28-29 weeks, 30-31 weeks, and 32 weeks	Evaluate cost-effectiveness of ACS in decreasing in-hospital morbidity of preterm infants with different gestational ages.	Decision tree model	2013	Cost per neonatal morbidity^a^	Hospital (provider)	Period of hospitalization until neonatal discharge	High (18/23)
Simpson 1995^28^	USA	National database from tertiary hospitals	ACS (not specified)	<28 weeks, 28-31 weeks.^b^	Estimate cost-effectiveness of ACS to improve health outcomes for premature infants, and to examine influence of birth weight and GA on cost-effectiveness estimates.	Decision tree model	1992	Costs; deaths averted, “Index Diseases” averted^a^	Not specified	Neonatal period until discharge from hospital	Moderate (16/23)
**Antenatal corticosteroids at 34 - <37 weeks’ gestation**											
Bastek 2012^33^	USA	Tertiary hospital setting	ACS (not specified)	34, 35, 36 weeks reported separately	Determine whether ACS is cost-effective in late-preterm infants at risk of delivery.	Decision tree model	2011	Cost per QALY	Single payer	Lifetime effects	High (21/23)
Gyamfi-Bannerman 2019^34^	USA	Multi-centre trial in tertiary hospital settings	ACS (betamethasone)	34 weeks 0 days to 36 weeks 6 days	To assess whether betamethasone compared with standard of care (without betamethasone) was cost-effective.	Cost-effectiveness analysis based on a randomized trial	2015	Cost per respiratory morbidity^a^	Third party funder	First 72 hours of neonatal period	High (20/22)
Rosenbloom 2020^32^	USA	Multi-centre trial in tertiary hospital settings	ACS (betamethasone)	34 weeks 0 days to 36 weeks 6 days	Compare betamethasone administration versus no betamethasone administration in patients at risk of delivery in the late-preterm period.	Cost-effectiveness analysis based on a randomized clinical trial	2017	Cost per QALY	Health sector	7.5 days (median duration of neonatal admission in the trial)	High (20.5/22)
**Antenatal corticosteroids in preterm birth (broad or unspecified gestation)**											
Johnson 1981^35^	USA	Tertiary hospital	ACS (betamethasone)	26-35 weeks	Determine whether prenatal glucocorticoid administration decreased the cost of newborn intensive care as well as mortality in infants born prematurely.	Hospital charges alongside a retrospective cohort study	1979	Charges; survival, total length of hospitalisation	Not specified	Period of hospitalization until neonatal discharge	Moderate (13/22)
Memirie 2019^36^	Ethiopia	Inpatient facility	ACS (betamethasone)	Preterm birth (not otherwise specified)	Examine cost-effectiveness of selected interventions (including ACS) in Ethiopian setting.	Cost-effectiveness analysis	2018	Cost per DALY averted	Provider	Not specified	High (19/22)
Michalow 2015^37^	South Africa	Multiple facility settings	Increased coverage of ACS (not specified)	Preterm birth (not otherwise specified)	Evaluate the impact and cost-effectiveness of selected interventions (including ACS) acknowledged to prevent stillbirths and maternal and newborn mortality, in South African setting.	Cost-effectiveness analysis	2014	Cost per LY gained	Not specified	Not specified	Moderate (14.5/22)

a health outcome details specified in Appendix S2.

b Also considered birthweight groups <2kg, <1.5kg.

**Table 2:** Characteristics of included studies assessing cost-effectiveness of tocolytics for management of preterm labour.

Study	Country	Care setting	Intervention	Study population	Aim	Design / analytic approach	Year of cost estimates	Type of evaluations (main outcomes)	Analytic viewpoint (perspective)	Time horizon (for effects)	CHEERS overall quality assessment
**Tocolytic only – 48-hour endpoint to facilitate ACS administration**											
Ferriols 2005^41^	Spain	Inpatient setting	Protocol A: Ritodrine as first-choice tocolytic vs Protocol B: Atosiban as first-choice tocolytic	Women at 23-33 weeks with primary onset of preterm labour	To conduct a pharmacoeconomic assessment of two tocolysis protocols to delay birth for 48 hours in the acute management of premature birth risk in gravid women.	Decision tree model	Not specified	Costs; Success (i.e. delivery delayed for 48 hours), therapeutic failures (i.e. interruption of treatment due to adverse effects or progression of labor)	Health system perspective	48 hours from intervention	High (17/22)
Guo 2011^43^	Canada	14 tertiary hospitals	Transdermal nitroglycerin (GTN) patch vs placebo	Women at 24 weeks 0 days to 32 weeks 0 days with primary onset of preterm labour	Determine cost-effectiveness of GTN for preterm labor	Cost-effectiveness analysis based on a randomised clinical trial	2003-04	Cost per case admitted to the NICU	Hospital (provider)	Period of hospitalization until neonatal discharge from NICU	High (18/22)
Hayes 2007^38^	USA	Not specified	Indomethacin vs Nifedipine vs Magnesium sulphate vs subcutaneous terbutaline	Women with primary onset of preterm labour (not otherwise specified)	To determine which of four tocolytics should be considered the agent of choice, based on the risk and costs of adverse events.	Decision tree model; cost-benefit analysis	2005	Costs; Adverse events were converted into costs and total costs compared.	Hospital (provider)	48 hours after diagnosis of labour	High (18.5/22)
Heinen-Kammemer 2003^39^	Germany	Inpatient setting	Atosiban vs Continuous fenoterol vs Bolus fenoterol vs Fenoterol with magnesium sulphate	Women with primary onset of preterm labour (not otherwise specified)	Determine which of four treatment alternatives is the most cost-effective form the perspective of statutory health insurance and nursing insurance.	Decision tree model	Not specified	Costs; the delay in giving birth at least 48 hours after the start of treatment, occurrence of adverse drug reactions	Payer perspective: statutory health insurance and statutory long-term care insurance	Observation period of 48 hours, extended to 10 days in the event of therapy failure; length of inpatient stay for unwanted drug effects outcome; five years for hearing impairment outcome.	Moderate (15.5/22)
Hruby 2004^40^	Czech Republic	Hospital pharmacy	Atosiban vs Fenoterol vs Hexoprenalin	Not specified	Evaluate cost of treating premature delivery with atosiban or beta-sympatomimetic drugs	"Pharmaco-economic model" based on a randomized, controlled clinical study. Apparent cost-benefit analysis.	Not specified	Costs; treatments for adverse effects for the next 72 h after the administration of the drugs were converted into costs, and total costs compared.	Health care payer perspective (medical insurance company).	Period of 18 and 48 h, treating adverse effects for 72 h after administering tocolytics.	Unable to assess^a^
Nijman 2019^42^	Netherlands, Belgium	19 facilities (seven secondary care and twelve tertiary care)	Nifedipine vs Atosiban	Women at 25 weeks 0 days to 33 weeks 0 days gestation in preterm labour	Compare the costs and effects of nifedipine and atosiban in women with a threatened preterm birth.	Cost-effectiveness analysis alongside randomised clinical trial	2013	Costs: a composite of adverse perinatal outcomes^b^	Societal	Neonatal period up to 6 weeks postpartum	High (22.5/23)
Wex 2009^44^	Germany	Multiple inpatient facilities	Atosiban vs Fenoterol	Women at 23 to 33 weeks’ gestation in preterm labour	Compare economic implications of tocolysis using atosiban or betamimetics, considering treatment efficacy and safety, as well as cost consequences of treatment of associated adverse events.	Cost-minimisation analysis; based on a systematic review of trials.	Not specified	Costs: efficacy in delaying preterm birth by at least 48 hours, frequency of maternal and foetal adverse events	Multiple perspectives: hospital (provider) perspective, payer perspective (unspecified), combined perspectives/	Outcomes during first 48 hours of hospitalisation	High (19.5/22)
Wex 2011^45^	Italy	Multiple inpatient facilities	Atosiban vs betamimetics (ritodrine, isoxuprine)	Not specified	Determine the cost-effectiveness of atosiban compared to betamimetics in the treatment of preterm labour within the Italian setting.	Cost-minimisation analysis; based on a systematic review of trials.	2010	Costs; efficacy in delaying preterm birth by at least 48 hours, frequency of maternal and fetal adverse events	Multiple perspectives: hospital (provider) perspective, payer perspective (unspecified), combined perspectives	Outcomes during first 48 hours of hospitalisation	High (19.5/22)
**Tocolytic only – acute and maintenance tocolysis**											
Ambrose 2004^51^	USA	Tertiary inpatient setting and outpatient setting	Maintenance subcutaneous terbutaline inpatient vs outpatient	Women from 24 to <34 weeks (for commencement of ongoing subcutaneous terbutaline after initial quiescence of primary onset preterm labour)	To compare pregnancy and economic outcomes of inpatient vs outpatient management of women with stabilized preterm labor treated with low-dose continuous subcutaneous terbutaline.	Total antepartum hospital, nursery, and outpatient charges considered in a retrospective cohort study.	Not specified	Charges^c^; Gestational age at delivery, Preterm delivery <37 weeks, Birth weight (g) >2500 g, NICU admission, Total nursery days	Not specified	Maternal antepartum and neonatal period until discharge from hospital post-delivery	Low (10/22)
Fleming 2004^49^	USA	Tertiary hospital setting for initial tocolysis and outpatient setting for ongoing tocolysis	Maintenance subcutaneous terbutaline vs nifedipine	Women who were: (1) singleton gestation, (2) prescribed nifedipine following an initial episode of preterm labor, (3) subsequent hospitalization for recurrent preterm labor at <34 weeks, (4) stabilized by tocolysis per attending physician's plan of treatment, and (5) outpatient tocolysis resumed with nifedipine or continuous subcutaneous terbutaline.	To compare gestational days gained and the associated costs of using oral nifedipine versus continuous subcutaneous terbutaline infusion for ongoing tocolysis in patients with recurrent preterm labor.	Medical costs considered in a retrospective cohort study	1999	Estimated costs; Days gained following recurrent preterm labor, delivery <35 weeks, neonatal outcomes (NICU, stillbirth, birthweight, others^a^)	Not specified	Maternal antepartum and neonatal period until discharge from hospital post-delivery	Low (9.5/23)
Flick 2010^50^	USA	Tertiary hospital setting for initial tocolysis, outpatient setting ongoing tocolysis	Maintenance subcutaneous terbutaline vs nifedipine	Women <35 weeks’ gestation, hospitalized for at least 24 hours, received preterm labor treatment, and had intact membranes, and were subsequently discharged to resume outpatient services with oral nifedipine or continuous subcutaneous terbutaline infusion	To examine pregnancy outcomes of women receiving oral nifedipine tocolysis following hospitalization for recurrent preterm labor symptoms versus outcomes of women having an alteration in treatment from nifedipine to continuous subcutaneous terbutaline.	Charges considered in a retrospective cohort study	Not specified	Chargese; prolonged pregnancy days, gestational age at delivery, birth weight, NICU days, nursery days	Not specified	Maternal antepartum and neonatal period until discharge from hospital post-delivery	Low (11/23)
Jakovljevic 2008^52^	Serbia, Montenegro	Single tertiary hospital	Ritodrine vs Fenoterol	Women at 26.6 ± 6.7 weeks in preterm labour	Investigate cost-effectiveness of two betamimetic agents (ritodrine and fenoterol) for treatment of preterm labor	Cost-effectiveness analysis based on a cohort study	2006	Costs; length of pregnancy (in weeks), prolongation of the pregnancy (in weeks), and score on modified Flanagan's quality-of-life scale for chronic diseases, measured after discharge from the hospital.	Third party funder	Maternal period of treatment and hospitalisation	Moderate (14.5/23)
Korenbrot 1984^56^	USA	Tertiary hospital	Beta-adrenergic tocolysis (terbutaline, isoxsuprine) vs no tocolysis	Women at 20 to 37 weeks gestation with preterm labour	Compare the inpatient charges, obstetrician and pediatrician fees, and outpatient high-risk obstetric follow up charges for treated mothers with costs that would have been incurred if they had not been treated and their infants had been born without gestational delay.	Cost-effectiveness analysis based on a retrospective cohort study	1981	Charges; proportion with arrested labour, extension of gestation, perinatal and neonatal survival rate	Not specified	Length of pregnancy until neonatal discharge	Low (10.5/22)
Lam 2001^46^	USA	Outpatient setting for the intervention after stabilisation in an inpatient setting.	Maintenance subcutaneous terbutaline vs oral tocolytics (terbutaline, magnesium, nifedipine, indomethacin or combination)	All women with twin gestations who experienced an initial episode of preterm labor which was treated with oral tocolysis, and subsequently hospitalized for recurrence of preterm labor symptoms at <35 weeks’ gestation.	To evaluate the clinical and cost-effectiveness of using continuous subcutaneous terbutaline versus oral tocolysis to treat recurrent preterm labor in twin gestations.	Total antepartum hospital, nursery, and outpatient charges considered in a retrospective cohort study.	Not specified	Chargese; Gestational days gained, gestational age at delivery, perinatal losses, nursery days, admissions to NICU and length of stay in NICU, birth weight, caesarean delivery.	Not specified	Maternal antepartum and neonatal period until discharge from hospital post-delivery	Low (10/23)
Lam 2003^47^	USA	United States; outpatient setting for the intervention after stabilisation in an inpatient setting.	Maintenance subcutaneous terbutaline vs oral tocolytics (terbutaline, magnesium, nifedipine, indomethacin or combination)	Women meeting the following criteria: 1) singleton gestation, 2) initial episode of preterm labor at greater than 20 weeks, 3) subsequent hospitalization for recurrent preterm labor at less than 35 weeks. Women who were stabilized and later discharged to home following recurrent preterm labor were eligible for study inclusion.	To compare the clinical and cost-effectiveness of utilizing continuous subcutaneous terbutaline versus oral tocolysis following recurrent preterm labor in women with singleton gestations.	Total antepartum hospital, nursery, and outpatient charges considered in a retrospective cohort study.	Not specified	Chargese; Gestational days gained, gestational age at delivery, perinatal losses, nursery days, admissions to NICU and length of stay in NICU, birth weight, caesarean delivery.	Not specified	Maternal antepartum and neonatal period until discharge from hospital post-delivery	Low (10.5/23)
Morales 1989^55^	USA	Tertiary hospital	Indomethacin vs Ritodrine	Pregnant women <32 weeks gestation	To compare, using a prospective randomised design, the relative efficacy of maternal/neonatal effects of indomethacin vs ritodrine hydrochloride.	Costs alongside randomised clinical trial	Not specified	Costs; effectiveness of tocolysis agent (time gained, time to stop contractions, contraction frequency, cervical dilation), maternal side effects, and neonatal outcomes.	Not specified	Period of hospitalisation until neonatal discharge	Low (9.5/22)
Morrison 2003^48^	USA	Outpatient setting for the intervention after stabilisation in an inpatient setting.	Maintenance subcutaneous terbutaline vs no intervention	Women <32 weeks’ gestation during recurrent preterm labour	Assess the effectiveness of ambulatory administration of continuous parenteral terbutaline to women at very high risk for early delivery (<32 weeks) compared with women who did not receive any therapy, in the home on an outpatient basis.	Newborn costs included in a retrospective cohort study	Not specified	Costs; adverse effects of terbutaline, pregnancy prolongation, maternal and neonatal morbidityd	Not specified	Maternal antepartum and neonatal period until discharged from hospital	Low (9.5/22)
Tomczyk 2015^54^	Poland	Tertiary hospital	IV followed by continuous oral fenoterol vs IV for 48-72 hours only	Women at risk of labour at 24-34 weeks gestation	To compare cost and effectiveness of fenoterol therapy in pregnant women at risk of preterm labour in the hospital for two consecutive years: 2012, when fenoterol was widely used, and 2013, when restrictions were introduced.	Cost analysis alongside a retrospective cohort study	Not specified	Cost of hospitalisation; mean week of delivery, mode of delivery, neonatal weight, delivery at term, APGARs, Hb and CRP after delivery, betamethasone and antibiotic administration	Not specified	Period of hospitalisation	Low (10/22)
Valdés 2012^53^	Chile	3 Maternal-fetal units at tertiary hospitals	Nifedipine (oral) vs Fenoterol (intravenous)	Women at 22 to 34 weeks’ gestation in preterm labour	Compare efficacy of nifedipine and fenoterol as a first-line tocolytic agent in the management of threatened preterm labor.	Cost-minimisation analysis alongside randomised clinical trial	2007-08	Total costs; Outcomes from the RCT included: Clinical, metabolic, hemodynamic endpoints, the gestational age upon recruitment, effectiveness of the assigned tocolytic, latency period, adverse effects, the incidence of preterm delivery and perinatal outcomes.	Not specified	Period of hospitalization until neonatal discharge	Low (10.5/22)
Weiner 1988^57^	USA	Tertiary hospital	Tocolysis (Ritodrine, terbutaline, or magnesium sulfate) vs bed rest	Women <34 weeks gestation with premature rupture of membranes	Determine the therapeutic efficacy, safety, and cost-effectiveness of tocolysis for preterm labor after membrane rupture.	Cost-effectiveness analysis alongside randomised clinical trial	Not specified	Costs; gestational age at delivery, birth weight, maternal or fetal infectious morbidity, respiratory distress syndrome, necrotizing enterocolitis, perinatal mortality	Not specified	Period of hospitalisation until neonatal discharge.	Moderate (14/22)

a Unable to fully assess study quality as only abstract was available.

b health outcome details specified in Appendix S2.

c Charges refer to patient costs (cost price of treatment with any additional charges to the patient).

**Table 3:** Characteristics of included studies assessing cost-effectiveness of antenatal corticosteroids and tocolytic therapy in combination.

Study	Country	Care setting	Intervention	Study population	Aim^a^	Design/analytic approach	Year of cost estimates	Type of evaluations (main outcomes)	Analytic viewpoint (perspective)	Time horizon (for effects)	CHEERS overall quality assessment
Mozurkewich 2000^60^	Canada	Outpatient setting for universal administration of corticosteroids without tocolytics, inpatient setting for testing strategies and tocolysis.	ACS (unspecified) + Tocolytic (unspecified) vs ACS only vs no intervention	Women in preterm labour at 24 to 34 weeks’ gestation	To compare the cost-effectiveness of nine strategies for the management of threatened preterm labour	Decision tree model	1999	Costs; number of RDS cases (with survival) per strategy, and number of neonatal deaths per strategy.	Third-party payer perspective	Period of hospitalisation until neonatal discharge or death of the newborn.	High (18/22)
Myers 1997^61^	USA	Tertiary hospital	ACS (unspecified) + Tocolytic (betamimetic) vs no intervention	Women in preterm labour at <37 weeks’ gestation	To determine the most cost-effective strategy for preventing RDS in the infants of women with preterm labour, comparing tocolysis and corticosteroids; amniocentesis and testing for fetal lung maturity, with treatment based on test results; and no treatment.	Decision analysis, Markov model	1996	Costs, cost per case of RDS averted	Hospital (provider)	7-day time frame (initial hospitalisation)	High (18.5/22)
van Baaren 2013^58^	Netherlands	Tertiary hospital	ACS (unspecified) + Tocolytic (nifedipine) vs no intervention	Women in preterm labour at 24 to 34 weeks’ gestation	To evaluate the cost- effectiveness of risk stratification with cervical length measurement and/or fetal fibronectin tests in women with threatened preterm labour between 24 and 34 weeks’ gestation.	Decision tree model	2011	Costs; Proportion of patients treated, perinatal death, a composite of adverse neonatal outcomes^b^	Health sector	Period of hospitalisation until neonatal discharge.	High (20.5/22)
van Baaren 2018^59^	Netherlands	Tertiary hospital	ACS (unspecified) + Tocolytic (nifedipine) vs no intervention	Women in preterm labour at 24 to 34 weeks’ gestation	To evaluate the cost-effectiveness of combining cervical-length measurement and fetal fibronectin testing in women with symptoms of preterm labor between 24 and 34 weeks’ gestation.	Decision tree model	2011	Costs; Proportion of patients treated, perinatal death, a composite of adverse neonatal outcomesg	Societal	Period of hospitalisation until neonatal discharge.	High (20.5/22)

a For this systematic review, only those arms (or comparisons) pertaining to ACS and tocolytic use were considered.

b health outcome details specified in Appendix S2.

Included studies were published between 1981 and 2019, and were conducted in high-income (31 studies), upper-middle income (3 studies), and low-income (1 study) countries. Five of the studies on ACS related to administration prior to 34 weeks’ gestation,^26^, ^27^, ^28^, ^29^, ^30^, ^31^ three were on its use in the late preterm period (34 to <37 weeks’ gestation),^32^, ^33^, ^34^ one studied both categories,^35^ and two did not specify (Table 1).^36^^,^^37^ Eight of the studies on tocolytics for managing preterm labour examined the use of tocolytics for facilitating ACS administration,^38^, ^39^, ^40^, ^41^, ^42^, ^43^, ^44^, ^45^ and twelve studies related to tocolytic use for acute and maintenance tocolysis without explicit consideration of ACS (Table 2).^46^, ^47^, ^48^, ^49^, ^50^, ^51^, ^52^, ^53^, ^54^, ^55^, ^56^, ^57^ The aim of the studies considering the combination of ACS and tocolytics (Table 3) was to examine different test-treatment strategies in the setting of preterm birth; and data relevant to ‘treatment only’ options were extracted.^58^, ^59^, ^60^, ^61^ Results are presented for each of these sub-categories.

### Antenatal corticosteroids

#### Preterm birth prior to 34 weeks’ gestation

Five studies examined cost-effectiveness of ACS prior to 34 weeks’ gestation, and were conducted in the United States of America (USA) (two studies), the United Kingdom (UK), the Netherlands and Brazil (Table 1).^26^^,^^28^, ^29^, ^30^, ^31^ Morales et al (1986) considered dexamethasone only,^30^ Ogata et al (2016) considered either betamethasone or dexamethasone,^26^ and three studies did not specify.^28^^,^^29^^,^^31^ Three studies used decision modelling techniques^26^^,^^28^^,^^31^ while two studies considered costs alongside a retrospective cohort study^29^ and a randomised controlled trial^30^ respectively. Ogata et al (2016) specified a provider perspective^26^ while the other four studies did not specify a perspective.^28^, ^29^, ^30^, ^31^ All five studies used a short-term time horizon for costs and outcomes (until neonatal discharge from hospital). Methodological quality was high for one study,^26^ moderate for three studies,^28^^,^^29^^,^^31^ and low for one study.^30^

The Ogata et al study in Brazil (2016) found that ACS significantly reduced most neonatal morbidity outcomes and hospitalisation costs in infants who survived hospitalisation, except for late-onset sepsis where the probability increased by 2·5% (Table 4).^26^ Simpson et al (1995) found that in USA hospital settings ACS reduced hospital costs, deaths, and specific neonatal morbidities (index cases) in all infants born <2kg, as well as in premature infants at 28 to 31 weeks. In premature infants <28 weeks, the ACS treatment group had fewer deaths but a greater number of index cases; however, ACS was still cost-saving in terms of hospital costs.^28^ In the Netherlands, Egberts et al (1992) found that ACS reduced deaths, cases of RDS, and costs per survivor, but more survivors meant total costs increased compared to no treatment (24300 DFL per extra survivor).^29^ In the USA in 1986, Morales et al reported ACS reduced costs, hospitalisation time, RDS cases, and IVH cases compared to no treatment.^30^ Mugford et al (1991) found that in the UK ACS reduced deaths, RDS cases, and costs compared to no treatment.^31^

**Table 4:** Summary of findings from cost-effectiveness studies of antenatal corticosteroids for preterm birth.

Study	Treatment options	Cost-effectiveness result(s)	Dominance / Cost-effectiveness	Summary of study conclusions
**Antenatal corticosteroids at <34 weeks’ gestation**				
Egberts 1992^29^	ACS (unspecified) Comparator: no treatment	ACS reduces RDS (OR 0.38 (0.24-0.60)) and mortality (OR 0.59 (0.47-0.75)) and costs 24300 Dfl per extra survivor compared to no treatment.	Cost-effective vs comparator	ACS reduces neonatal mortality and RDS, but increases total hospital time and costs
Morales 1986^30^	ACS (dexamethasone) Comparator: no treatment	Reduced incidence of RDS (51% vs 25%) and intraventricular haemorrhage (27% vs 11%), reduced hospitalisation length (38 vs 22 days per infant). Reduced average cost per patient from $27,600 versus $10,300.	Dominant vs comparator	Statistically significant reduction in the incidence of respiratory distress syndrome and intraventricular haemorrhage, time of hospitalisation, and average cost per patient. No difference the rate of chorioamnionitis and neonatal sepsis, and no statistically significant difference in the incidence of severe intraventricular haemorrhage, necrotizing enterocolitis, or mortality.
Mugford 1991^31^	ACS (not specified) in two population subgroups (<35 weeks GA; <31 weeks GA) Comparator: no treatment	*<35 weeks GA:* 2.5 deaths and 6.2 RDS cases averted per 70 infants. £394 saved per infant, and £634 saved per survivor. <31 weeks GA: 2.6 deaths averted, 0.4 additional RDS cases per 70 infants. £422 additional costs per infant, and £880 saved per survivor.	Dominant vs comparator	Use of ACS for women with gestations up to 35 weeks would have reduced the number of cases of RDS and the number of deaths, and reduced the costs of care. Use of ACS for the <31 weeks GA subgroup only would have increased total costs because of the greater cost of caring for babies who would have survived, but total cost per survivor would reduce.
Ogata 2016^26^	ACS (betamethasone or dexamethasone) Comparator: no treatment	US$3413 cost savings in hospital costs per patient and reduced newborn morbidity or no significant difference against 16 outcome measures.	Dominant vs comparator	ACS was dominant compared to no treatment. Morbidity outcomes significantly decreased with ACS included advanced resuscitation in delivery room, use of surfactant, mechanical ventilation, blood transfusion, PIVH grades III and IV. The model was stable to sensitivity analysis. ACS was associated with a non-significant increased incidence of late-onset sepsis in the study population.
Simpson 1995^28^	ACS (not specified) in 3 population subgroups Comparator: no treatment	*Birth weight <2kg*: 4·4 deaths and 12·1 index disease cases averted, and US$326,200 combined hospital and physician costs saved per 100 infants *<28 weeks GA*: 16·8 deaths averted, 9·1 additional index disease cases, and US$467,700 USD saved per 100 infants *28-31 week GA*: 2·9 deaths and 16·6 index disease cases averted, and US$317,200 saved per 100 infants	Dominant vs comparator	ACS both improves health outcomes and yields cost savings. Sensitivity analysis in the birth weight <2kg population tested hospital only costs, or hospital plus 15% of physician charges, and still found cost savings.
**Antenatal corticosteroids at 34 - <37 weeks’ gestation**				
Bastek 2012^33^	ACS (not specified) in 3 population subgroups Comparator: no treatment	*34 weeks*: US$62,888·25 per QALY *35 weeks*: US$64,425·67 per QALY *36 weeks*: US$64,793·71 per QALY	Cost effective at threshold of US$100,000/QALY	Administration of ACS to women at risk of imminent delivery at 34-36 weeks’ gestation could significantly reduce the cost and acute morbidity associated with late preterm birth. While ACS was the consistently dominant strategy for acute respiratory outcomes, all models were sensitive to changes in probabilities and utilities associated with chronic respiratory disease.
	ACS (not specified) partial course in 3 population subgroups Comparator: no treatment	34 weeks: US$131,233·39 per QALY 35 weeks: US$133,117·42 per QALY 36 weeks: US$133,654·76 per QALY	Not cost effective at threshold of US$100,000/QALY	
Gyamfi-Bannerman 2019^34^	Betamethasone Comparator: no treatment	US$23,986 cost saving per case of respiratory morbidity averted	Dominant	Antenatal betamethasone treatment associated with a statistically significant decrease in health care costs and with improved outcomes; thus, this treatment may be an economically desirable strategy.
Rosenbloom 2020^32^	Betamethasone Comparator: no treatment	US$88m cost increase (US$1,780m vs US$1,692m) and decrease of 11 QALYs (5,405 vs 5,416) per 270,000 live births	Dominated by withholding treatment	Withholding betamethasone dominated betamethasone administration and was cost-saving, i.e. less costly and more effective. If betamethasone were provided free-of-charge (i.e., $0 cost for administration), withholding administration was still more effective and less costly.
**Antenatal corticosteroids in preterm birth (broad or unspecified gestation)**				
Johnson 1981^35^	ACS (betamethasone) Comparator: No treatment	Birth weight 750-999g ACS 89% survival, comparator 64% survival^a^ *Birth weight 1000-1249g:* ACS 88% survival, comparator 40% survival^a^ *Birth weight 1250-1499g:* ACS $17069±2442 vs comparator $24553±2379 in hospital charges^a^ Birth weight 1500-1749g: ACS $12012±1338 vs comparator $18207 ±3021 in hospital charges^a^	May be cost-effective; dominant over multiple birthweight categories combined	Infants whose mothers received two doses of betamethasone had a significantly lower mortality in the two smallest birthweight categories (750-999g, 1000-1249g). Infants in both treated and untreated groups with birth weights between 1250 and 1999g (30-33w gestation) had similar survival. Betamethasone treatment did not cause a statistically significant difference in hospital charges between 750-1249g (27-29 weeks gestation). However, infants with birth weights between 1250 and 1749g (30-32 weeks gestation) whose mothers received betamethasone had significantly lower total hospital charges.
Memirie 2019^36^	Betamethasone (20% increase in coverage) Comparator: no treatment (0% ACS coverage)	US$98 per DALY averted	Cost-effective	ACS is highly cost effective compared to no treatment.
Michalow 2015^37^	100% coverage of ACS (unspecified) Comparator: 20% coverage of ACS (unspecified)	$37 per LY saved	Cost-effective	Antenatal corticosteroids are highly cost-effective.

a Only results with p-value <0.05 reported.

#### Preterm birth at 34 to <37 weeks’ gestation

All three studies on ACS cost-effectiveness at 34 to <37 weeks’ gestation were undertaken in the USA (Table 1).^32^, ^33^, ^34^ Bastek et al (2012) used a literature review to construct a decision model considering ACS use from a single payer perspective.^33^ The other two studies used outcomes related to betamethasone use from the Antenatal Late Preterm Steroids (ALPS) trial:^62^ Gyamfi-Bannerman et al (2019) used a third-party funder perspective,^34^ while Rosenbloom et al (2020) used a health sector perspective.^32^ Bastek et al examined a lifetime horizon for costs and effects,^33^ while the other two studies used short time horizons – the first 72 hours^34^ or first 7·5 days of the neonatal period,^32^ respectively. All three studies were assessed as high methodological quality.

Bastek et al reported that the ICER for a full course of ACS (compared to no ACS) favoured the full course of ACS at 34, 35, and 36 weeks using a threshold of $100,000/QALY; a partial course of ACS was not cost-effective (Table 4).^33^ When comparing ACS to no ACS at 34 weeks alone, the ICER was $62,888⋅25/QALY, compared to $64,425⋅67/QALY at 35 weeks, and $64,793⋅71/QALY at 36 weeks in the base case – however, these were not robust across all variations of acute and chronic disease distribution. Sensitivity analyses restricted to distributions associated with acute respiratory disease demonstrated 95% confidence in ACS willingness-to-pay thresholds of >$64,677 at 34 weeks, >$65,700 at 35 weeks, and >$65,819 at 36 weeks. Gyamfi-Bannerman et al concluded that compared to placebo, betamethasone was more effective and decreased total mean costs for each woman-infant pair.^34^ Rosenbloom et al used the same trial data as Gyamfi-Bannerman et al and reported that betamethasone was dominated by no ACS.^32^ This can be attributed to Gyamfi-Bannerman et al costing the primary trial outcome only (a composite of neonatal respiratory treatment or stillbirth or neonatal death in the first 72 hours after birth), while Rosenbloom et al considered costs of additional outcomes (neonatal hypoglycaemia, which increased with betamethasone) alongside RDS and transient tachypnoea of the newborn (TTN), and derived utilities for each outcome from the literature to calculate QALYs. They reported ACS as being slightly more expensive and generating less QALYs than placebo.

#### Preterm birth (broad or unspecified gestation)

One study from the USA by Johnson et al (1981) examined ACS use (betamethasone) from 26 to 35 weeks’ gestation considering costs alongside a retrospective cohort study.^35^ Newborn effects until discharge from hospital were considered, though the perspective was not specified. Methodological quality was assessed as moderate. The authors reported a significantly lower mortality in the two smallest birthweight categories (750-999g, 1000-1249g) without statistically significant difference in hospital charges. Conversely, infants with birth weights between 1250 and 1749g (30-32 weeks’ gestation) incurred significantly lower hospital charges despite no difference in mortality, suggesting ACS is dominant when birth-weight categories are combined.

Two other studies conducted in Ethiopia and South Africa examined ACS use in preterm birth without specifying the gestational age range, using the Lives Saved tool (LiST) for cost-effectiveness analysis.^36^^,^^37^ The study in Ethiopia (high methodological quality) considered betamethasone and used a provider perspective, while the South Africa study (moderate methodological quality) did not specify either of these. Neither study reported the time horizon. Memirie et al (2019) found that increasing coverage of ACS in preterm labour by 20% in Ethiopia was highly cost-effective at $98 per DALY averted.^36^ Michalow et al (2015) found that increasing coverage of ACS from 20% to 100% in South Africa was highly cost-effective at $37 per life-year saved.^37^

### Tocolytics

#### Tocolytics to facilitate ACS administration

Eight studies assessed cost-effectiveness for tocolytics when used to prolong pregnancy for at least 48 hours, of which seven explicitly stated this was to facilitate ACS administration^38^^,^^39^^,^^41^, ^42^, ^43^, ^44^, ^45^ – the remaining study (abstract only) did not specify the reason (Table 2).^40^ All were conducted in high-income countries (Belgium, Canada, Czech Republic, Germany, Italy, Netherlands, Spain, and USA). Three studies conducted a cost-effectiveness analysis alongside a randomised trial,^40^^,^^42^^,^^43^ three studies constructed decision tree models using cost and outcome estimates from the literature,^38^^,^^39^^,^^41^ and two studies by the same group conducted cost-minimisation analyses alongside a systematic review.^44^^,^^45^ Analytical perspective varied between studies, including societal,^42^ hospital,^38^^,^^43^ health system,^41^ health insurance company,^39^^,^^40^ and multiple perspectives (hospital, payer and combined hospital and payer).^44^^,^^45^ Time horizons were generally short-term for both costs and outcomes – most studies focused on the 48 hours from time of hospitalisation or commencement of tocolysis.^38^, ^39^, ^40^, ^41^, ^42^, ^43^, ^44^, ^45^ One study examined outcomes until neonatal discharge from neonatal intensive care unit (NICU),^43^ one study was until six weeks postpartum,^42^ and one study considered hearing loss up to five years of age.^39^ Methodological quality was generally high (six studies) with one study of moderate quality; one study could not be fully assessed (abstract only).^40^

Most studies compared types of tocolytic agents and administration methods; only one study compared tocolysis with placebo, suggesting that transdermal GTN patches may be dominant with lower NICU admissions and associated costs (Table 5).^43^ Of the five studies comparing atosiban to different betamimetics (ritodrine, fenoterol, fenoterol with magnesium sulphate, hexoprenaline, isoxuprine), findings were mixed – two studies found atosiban to be equivalent to the comparator,^39^^,^^40^ two studies by the same lead author in different country settings (Italy and Germany) found that atosiban achieved equal effectiveness but at less cost than a betamimetic due to its superior safety profile,^44^^,^^45^ and one study concluded that ritodrine was more cost-effective as a first-line tocolytic than atosiban.^41^ One study comparing nifedipine and atosiban concluded that, in singleton pregnancies, nifedipine generated lower costs due to fewer NICU admissions; in multiple pregnancies, nifedipine was more effective and less costly.^42^ One study compared four agents (indomethacin, nifedipine, subcutaneous terbutaline, magnesium sulphate) and found indomethacin to be dominant in the base case, with nifedipine dominant in sensitivity analyses.^38^

**Table 5:** Summary of findings from cost-effectiveness studies of tocolytics for preterm labour.

Study	Treatment options	Cost-effectiveness result(s)	Dominance / Cost effectiveness	Summary of study conclusions
**Tocolytic only – 48 hour endpoint to facilitate ACS administration**				
Ferriols 2005 ^41^	Protocol A: Ritodrine as first-choice tocolytic agent to delay birth for 48 hours	€194 per effectiveness unita	Most cost-effective	Ritodrine as first-choice tocolytic agent (Protocol A) is more cost effective than Atosiban.
	Protocol B: Atosiban as first-choice tocolytic agent to delay birth for 48 hours	€632 per effectiveness unita	-	
Guo 2011 ^43^	Transdermal GTN patch	67·6% NICU admission avoided rate; Average cost per infant: CAN$13,397	Dominant	GTN arm was the dominant strategy, with both lower cost and higher NICU admission avoided rate compared to the placebo arm.
	Placebo patch	60·8% NICU admission avoided rate; Average cost per infant: CAN$18,427	-	
Hayes 2007 ^38^	Indomethacin for 48 hours	US$15·40 per patient	Dominant	Based on existing evidence of equal efficacy, indomethacin was found to be the dominant strategy for risk of adverse events and costs. Sensitivity analysis testing lowest and highest reported rates of adverse events indicated that nifedipine may be dominant over indomethacin which could indicate equivalence; however, each was superior to terbutaline.
	Subcutaneous terbutaline for 48 hours with monitoring	US$399·02 per patient	-	
	Nifedipine for 48 hours	US$16·75 per patient	Dominant in sensitivity analysis	
	Magnesium sulphate for 48 hours with monitoring	US$197·90 per patient	-	
Heinen-Kammemer 2003 ^39^	Atosiban up to 48 hours	€9,890 per successfully treated patient	-	By converting efficacy and adverse events into costs, therapy with fenoterol as a bolus dose was the most cost effective of the 4 options. However, sensitivity analysis indicated no robustness in the model.
	Fenoterol up to 48 hours	€1,1397 per successfully treated patient	Most cost-effective option	
	Bolus fenoterol up to 48 hours	€7,013 per successfully treated patient	-	
	Fenoterol with magnesium sulphate up to 48 hours	€8,972 per successfully treated patient	-	
Hruby 2004 ^40^	Atosiban treatment for up to 18 or 48 hours	*≤ 18 hours*: 21,914·5-21,974·4 CZK *≤ 48 hours*: 43,082·5-43,142·4 CZK	Dominated by alternative treatments	By presuming efficacy in delaying labour, in case of a shorter administration period (up to 18 hours): overall hospitalisation costs are comparable for administration of atosiban and beta-sympatomimetic drugs (fenoterol or hexoprenalin) when adverse events are converted into costs. In case of longer administration periods (more than 18 hours): overall hospitalisation costs are higher for administration of atosiban than beta-sympatomimetic drugs when adverse events are converted into costs. Overall costs increase as the duration of atosiban administration increase.
	Fenoterol treatment for up to 18 or 48 hours	*≤ 18 hours*: 19,878·7-22,661·4 CZK *≤ 48 hours*: 19,960·3-23,150·7 CZK	-	
	Hexoprenalin treatment for up to 18 or 48 hours	*≤ 18 hours*: 19,942·9-21,974·4 CZK *≤ 48 hours*: 20,131·3-23,574·0 CZK	-	
Nijman 2019 ^42^	Nifedipine for up to 48 hours in 2 population subgroups *Comparator*: intravenous atosiban for up to 48 hours of uterine quiescence	Singleton pregnancies: mean cost difference -€8479 (95% CI: -€14,327 to -€2016) Multiple pregnancies: mean cost difference -€12,044 (95% CI: -€21,607 to -€1671)	Dominant vs comparator	The trial found a non-significant difference in effectiveness for the composite primary outcome (singleton and multiple pregnancies). Mean costs per patient were significantly lower in the nifedipine group compared to the atosiban group for both singleton and multiple pregnancies. The main reason costs of atosiban were higher was that more neonates were admitted to the NICU.
Wex 2009 ^44^	Atosiban for 18 or 48 hours using 3 cost perspectives Comparator: continuous intravenous fenoterol for 18 or 48 hours	*Combined perspective*: cost savings of €226 for 18 hours of tocolysis; €71 for 48 hours *Payer perspective:* cost savings of €423 per patient. *Hospital perspective:* cost savings of €259 for 18 hours, €105 for 48 hours of tocolysis.	Dominant vs comparator	Atosiban is cost saving versus betamimetics in the treatment of preterm labour from the payer, hospital, and combined perspectives. Effectiveness estimates were based on three double-blinded, placebo-controlled trials which found identical efficacy in delaying preterm birth by at least 48 hours between atosiban and betamimetics. Cost savings stem from the superior safety profile of atosiban. Sensitivity analysis including all six identified RCTs likewise found no significant difference in effectiveness and that atosiban was cost-saving compared to fenoterol.
	Atosiban for 18 or 48 hours using 3 cost perspectives Comparator: bolus intravenous fenoterol for 18 or 48 hours	*Combined perspective:* cost savings of €211 for 18 hours of tocolysis; €21 for 48 hours *Payer perspective:* cost savings of €423 per patient. *Hospital perspective:* found cost savings of €244 for 18 hours, €55 for 48 hours of tocolysis.	Dominant vs comparator	
Wex 2011 ^45^	Intravenous atosiban up to 48 hours Comparator: Intravenous betamimetics up to 48 hours (Ritodrine, Isoxuprine)	Atosiban had similar efficacy and fewer adverse events than betamimetics. Cost savings per patient were €425 for 18 hours and €316 for 48 hours vs ritodrine; €429 for 18 hours and €326 for 48 hours versus isoxuprine from the combined (payer and hospital) perspective.	Dominant vs comparator	Owing to its superior safety profile, atosiban is cost-saving versus betamimetics in the treatment of preterm labour in Italy from the payer's, hospital's and combined perspectives.
**Tocolytic only – acute and maintenance tocolysis**				
Ambrose 2004 ^51^	Inpatient continuous subcutaneous terbutaline (SQT) to maintain tocolysis after an acute episode of preterm labour Comparator: Outpatient continuous SQT with nursing surveillance	Earlier gestational age at delivery (34·1±2·9 vs 35·8±1·9 weeks, p<0·001) Higher preterm birth rate (86·7% vs 74·4%, p=0.043) Higher overall costs (US$56,089± 47,944 vs US$25,540±25,847, p<0·001)	Dominated by comparator	Outpatient management of SQT was associated with better pregnancy outcomes and cost less than inpatient management. Outpatient SQT is dominant compared to inpatient management.
Fleming 2004 ^49^	Outpatient nursing services with nifedipine for recurrent preterm labour Comparator: Continuous outpatient subcutaneous terbutaline (SQT) with nursing services	Earlier GA at delivery (35·7±3·1 weeks versus 36·6±2·1 weeks, p=0·004) Higher healthcare utilization costs (US$37,040±47,518 versus US$26,546±25,386, p=0·014)	Dominated by comparator	Treating recurrent preterm labour with SQT versus oral nifedipine resulted in a later gestational age at delivery, improved neonatal outcomes, and increased cost-effectiveness. SQT is dominant compared to oral nifedipine.
Flick 2010 ^50^	Outpatient surveillance with nifedipine for recurrent preterm labour Comparator: Continuous outpatient subcutaneous terbutaline (SQT) with surveillance	More likely to deliver at <35 weeks (28·0% versus 13·8%), weigh <2500 g (32·9% versus 20·3%), and require a stay in the neonatal intensive care unit (34·0% versus 23·1%), all p<0·001. Higher costs (US$32,857±48,568 versus US$18,113±25,408, p<0·001)	Dominated by comparator	SQT delayed delivery further compared to oral nifedipine and increased gestational age at delivery, decreased number of NICU admissions, low birth weights, and overall costs.
Jakovljevic 2008 ^52^	Ritodrine (with verapamil and diazepam)	11·6±7·1 weeks prolongation of pregnancy; cost of 4,181·96 ±12,069·83 CSD per week of pregnancy prolongation gained	-	Prolongation of pregnancy was significantly longer in the fenoterol group than in the ritodrine group, and the mean duration of hospitalization was shorter. Treatment with fenoterol was less costly and more cost-effective than the treatment with ritodrine, but the difference in cost- effectiveness was not statistically significant due to low costs of hospitalisation and human labour in Serbian health system.
	Fenoterol (with verapamil and diazepam)	12·7±8·4 weeks prolongation of pregnancy; cost of 3,345·51±7,668·04 CSD per week of pregnancy prolongation gained	Dominant (non-significant)	
Korenbrot 1984 ^56^	Beta-adrenergic tocolysis (terbutaline, isoxsuprine) Comparator: No tocolysis	*20-25 weeks:* gestation extension of 14±1.1 weeks; improved survival rate from 20% to 80%. Costs approximately $5000 lower in treatment group (not statistically significant). *26-33 weeks*: gestation extension from 6.6±1.5 to 4.3±0.5; improved survival rates from 75-95% to 89-97%. Costs between $3730-23850 lower. Both effect and cost differences reduced over these ranges as gestational age increased. *34-37 weeks*: gestation extension from 3.9±0.5 (34-35 weeks) to 2.3±0.7 (36-37 weeks); survival and costs did not differ significantly.	Dominant (may not be statistically significant)	Treatment between 26 and 33 weeks was cost-effective. After 33 weeks there was no significant difference in survival or costs with or without treatment. The number of mothers not treated between 20-25 weeks was too small to permit statistical significance of results.
Lam 2001 ^46^	Continuous outpatient subcutaneous terbutaline (SQT) for recurrent preterm labour in twin gestations Comparator: Oral tocolytics (terbutaline, magnesium, nifedipine, indomethacin or combination)	Increase of 4.5 gestational days (35·2±2·0 versus 34·5±2·3, p<0·001), higher birth weight (2343±493g versus 2207±523g, p<0·001), and fewer NICU days (17·3±16·1 versus 20·8±17·4, p=0·009) US$17,109 total average cost saving (US$38,152±50,822 versus 55,261±60,932, p<0·001) per infant	Dominant	Infants of the SQT group had greater gestational age at delivery, higher birth weights, and less frequent neonatal intensive care unit admission. Charges for antepartum hospitalization and nursery were significantly less in the SQT group, while charges for outpatient services were less for the oral group. Mean total charges showed a cost saving for SQT.
Lam 2003 ^47^	Continuous outpatient subcutaneous terbutaline (SQT) for recurrent preterm labour Comparator: Oral tocolytics (terbutaline, magnesium, nifedipine, indomethacin or combination)	Higher gestational gain (33·9±19·0 days vs 28·4±19·8 days, p<0·001) per patient US$5,286 average cost saving (US$16,649±21,701 vs US$21,935±33,107, p<0·017) per patient	Dominant	The SQT group had more gestational gain following recurrent preterm labor than the oral tocolytics group and had lower average charges for antepartum hospitalisation and nursery. However, average outpatient charges were lower for the oral tocolytics group. SQT appears to be a dominant strategy compared with oral tocolytics.
Morales 1989 ^55^	Indomethacin (suppository, oral) ± magnesium sulphate *Comparator*: Ritodrine (IV) ± magnesium sulphate	Equally successful in stopping uterine contractions and delaying delivery for at least 48 hours in 94% and 83% of their respective uses. Cost savings of $33 per patient compared to $560 per patient (drug and monitoring costs only)	Dominant	Both tocolytics were equal in effect. Indomethacin preferable in side effect profile, driving lower cost of drug administration.
Morrison 2003 ^48^	Continuous outpatient subcutaneous terbutaline (SQT) after recurrent preterm labour Comparator: No outpatient tocolytic therapy after stabilisation in hospital	Better neonatal outcomes: gestational age at delivery more than 37 weeks (53% vs 4%), percentage delivered at less than 32 weeks (0% vs 47%), pregnancy prolongation (49·8 ± 19·2 days vs 24·5 ± 12·8 days); all p<0·001. Lower total cost for newborn care ($6,995±14,822 vs $62,033±89,978, p<0·002)	Dominant	Gestational age at delivery >37 weeks delivery <32 weeks and pregnancy prolongation were all significantly better in the SQT group. Cost savings in the SQT group arise from lower total number of maternal hospital days and shorter duration of NICU stay. SQT appears to be a dominant strategy compared with no outpatient tocolytic therapy following stabilisation.
Tomczyk 2015 ^54^	IV followed by continuous oral fenoterol Comparator: IV fenoterol for 48-72 hours only	Perinatal outcomes (AGPAR score and neonatal weight) were comparable. Cost savings were not significant (4334,700PLN vs. 5232,470PLN, p= 0.533)	No statistically significant result	No significant differences in success of tocolysis, maternal or neonatal outcomes, costs.
Valdés 2012 ^53^	Nifedipine for management of threatened preterm labour Comparator: Intravenous fenoterol	Lower success rate to obtain tocolysis when used as a first-line agent (80·3% vs. 90·9%, p=0·0001). Smaller proportion of adverse drug reactions (19% vs 57·8%, p=0·0001). No significant difference in costs (US$588±47·0 vs 951±277·6, not significant).	No statistically significant result	The study did not demonstrate either clinical or economic superiority of any of the two options. Nifedipine failed more frequently to obtain tocolysis when used as a first-line agent, while women treated with fenoterol had more drug adverse events. While the total healthcare cost with fenoterol was higher than with nifedipine, it was not statistically significant. However, the use of fenoterol was more burdensome in terms of bed-days, supplies, medications and specialist consultations.
Weiner 1988 ^57^	Intravenous tocolysis (ritodrine, terbutaline, magnesium sulfate) Comparator: bed rest	<28 weeks: significant increase in intrauterine time (232.8 ± 312 vs 53.4 ± 87) but no identifiable perinatal benefit in the tocolysis arm. Costs per survivor were higher in the tocolysis arm ($118206±42172 vs $82871±30650) >28 weeks: No significant increase in intrauterine time and no identifiable perinatal benefit. Differences in cost per survivor were not significant ($22670±15195 vs $23302±22770)	Unclear	Because tocolysis does not improve perinatal outcome and can itself be associated with major maternal morbidity, it should be avoided after 28 weeks' gestation. Before 28 weeks' gestation tocolysis may increase intrauterine time, but the benefit of this is not clear.

#### Acute and maintenance tocolysis

Twelve studies examined tocolytic use for acute and maintenance tocolysis (Table 2). Most studies compared types of tocolytic agents and administration methods, though two studies from the USA in the 1980’s compared tocolysis with no tocolysis.^56^^,^^57^ Five studies – all conducted in the USA between 2001 and 2009 – considered acute and maintenance tocolysis in women with recurrent preterm labour,^46^, ^47^, ^48^, ^49^, ^50^ three studies from the USA, and Serbia and Montenegro, considered acute and maintenance tocolysis in preterm labour,^52^^,^^56^^,^^57^ two studies from Chile and the USA examined acute tocolysis with subsequent surveillance,^53^^,^^55^ one study compared intravenous followed by continuous oral fenoterol with intravenous fenoterol for 48-72 hours only,^54^ and one study examined maintenance with subcutaneous terbutaline in an inpatient versus outpatient setting.^51^ Five studies used patient data from the same Matria Healthcare database^46^^,^^47^^,^^49^, ^50^, ^51^ and seven studies conducted cost-effectiveness analyses based on prospective cohort studies or trials.^48^^,^^52^, ^53^, ^54^, ^55^, ^56^, ^57^ One study took a third party funder perspective,^52^; no other study described the perspective.^46^, ^47^, ^48^, ^49^, ^50^, ^51^^,^^53^, ^54^, ^55^, ^56^, ^57^ Time horizons were short-term, with all studies examining an endpoint of initial discharge from hospital. Methodological quality was generally low (10 studies) with two studies of moderate quality – most provided no perspective or decision model, and few reported assumptions or performed uncertainty analyses.

Of the five studies examining tocolysis for recurrent preterm labour, subcutaneous terbutaline was found to be the dominant intervention in significantly increasing gestational age at birth, decreasing neonatal morbidity and decreasing overall costs when compared to oral tocolytics,^46^^,^^47^ oral nifedipine,^49^^,^^50^ or no outpatient therapy following stabilisation (Table 5).^48^

Ambrose et al (2004) found that outpatient subcutaneous terbutaline was dominant compared to inpatient administration, with later gestation ages at birth, lower preterm birth rates, and lower overall costs.^51^ Valdés et al (2012) found that while nifedipine and fenoterol achieved similar tocolytic effects, nifedipine was more likely to fail as a first-line agent, though fenoterol had more adverse reactions; costs were equivalent for both drugs.^53^ Jakovljevic et al (2008) found that when comparing acute and maintenance regimens using ritodrine and fenoterol (both betamimetics), the difference in tocolysis time and costs were not different (generating similar incremental cost-effectiveness ratios), although they suggested these findings might be specific to the Serbian healthcare context.^52^ Tomczyk et al (2015) found no significant differences in costs or effects between continuous fenoterol and fenoterol for 48-72 hours only.^54^ Morales et al (1989) found that indomethacin and ritodrine were equivalent in efficacy, but ritodrine was significantly more expensive than indomethacin ($33 per patient vs $560 per patient in drug and monitoring costs alone).^55^ Of the two studies comparing tocolysis with no tocolysis, Korenbrot et al (1984) found that acute and maintenance betamimetic tocolysis was dominant between 26-33 weeks compared to no tocolysis, with better outcomes and lower costs;^56^ Weiner et al (1988) did not find any significant difference in costs or outcomes between aggressive tocolysis (ritodrine, terbutaline, or magnesium sulphate) and oral maintenance therapy compared to bed rest.^57^

### Cost-effectiveness studies of ACS and tocolytics in combination

Four studies were identified which compared different test-treatment combination strategies for preterm labour; data were extracted and compared for strategies that combined ACS and tocolytics without testing (“treat all”), and no treatment or testing (“treat none”);^58^, ^59^, ^60^, ^61^ one study also compared these options to ACS only.^60^ Two studies performed decision modelling and cost-effectiveness analysis based on the APOSTEL-I and APOSTEL-II trials which compared nifedipine to placebo. One study specified use of effectiveness data for betamimetics, and one study based their analysis on a systematic review of multiple tocolytics. No study specified which type of ACS was used. All four studies were conducted in high-income countries (Netherlands,^58^^,^^59^ United States,^61^ Canada^60^) and constructed decision models from published data. All studies used short time horizons, such as hospitalisation until discharge 58, 59, 60 or up to 7 days.^61^ Analytical perspectives were third-party payer,^60^ provider,^61^ health sector,^58^ and societal.^59^ Methodological quality was high for all four studies.

Mozurkewich et al (2000) found that “treat none” was both more expensive and had higher rates of morbidity and mortality compared to “treat all” (ACS and tocolytics) or universal administration of ACS without tocolysis. Universal ACS only was the least expensive option, but resulted in more deaths and cases of RDS than universal ACS with tocolysis.^60^ Myers et al (1997) also found that “treat all” was dominant compared to “treat none” at probabilities of RDS > 2%, with lower costs and better outcomes.^61^ Van Baaren et al (2013 and 2018) found that “treat all” had increased costs but fewer deaths and adverse outcomes compared to “treat none” in two studies using different cost perspectives.^58^^,^^59^ Table 6 shows cost per patient treated, perinatal mortalities and adverse outcomes reported separately for each intervention in three studies,^58^, ^59^, ^60^ and cost-effectiveness ratios in one study.^61^

**Table 6:** Summary of findings from cost-effectiveness studies of antenatal corticosteroids and tocolytic therapy in combination.

Study	Treatment options	Cost-effectiveness result(s)	Dominant strategy^a^	Summary of study conclusions
Mozurkewich 2000 ^60^	Tocolytics and corticosteroids (“treat all”) Not to treat any women ("treat none") Treat all with outpatient corticosteroids, no tocolytics (“ACS only”)	50 RDS cases and 38 deaths per 1000 patients US$14,900 per patient, 102 RDS cases and 55 deaths per 1000 patients US$14,100 per patient, 61 RDS cases and 40 deaths per 1000 patients US$12,000 per patient	More costly, more effective Dominated by “ACS only” Less costly, more effective than “treat none”	Universal administration of outpatient corticosteroids was the least expensive option, but resulted in more cases of respiratory distress syndrome (RDS) and deaths than the “treat all” option. Treating all patients resulted in the fewest cases of RDS and deaths but the greatest costs. The “treat none” strategy resulted in more RDS cases, more deaths, and higher costs, so was dominated by both the “treat all” and “ACS only” options.
Myers 1997 ^61^	Betamimetics and antenatal steroids ("treat all") assuming varying probabilities of respiratory distress syndrome (RDS) Comparator: No intervention (“treat none”)	*Pr(RDS)=25%:* 81 vs 129 RDS cases per 1000 patients; average cost $14,493 vs $20,485 per patient *Pr(RDS)=12.5%:* 40 vs 64 RDS cases per 1000 patients; average cost $10,014 vs $12,585 per patient *Pr(RDS)=1%:* 3 vs 5 RDS cases per 1000 patients; average cost $5894 vs $5124 per patient. ICER of $2,916,016 per RDS case prevented compared to “treat none”	Dominant if probability of RDS is >2%	“Treat all” was cost saving and more effective compared with no treatment at probabilities of RDS above 2%. It may be cost-effective to use no treatment at probabilities of RDS less than 2%. Sensitivity analysis indicated “Treat all” was more cost effective as the costs of RDS and preterm birth increased.
van Baaren 2013 ^58^	Tocolysis and steroids with tertiary centre transfer (“treat all” reference strategy) Comparator: No treatment (“treat none”)	Reduction in perinatal mortality of 0·6 (95%CI: -1·7 to 2·9) per 1000 women. Reduction in number of poor outcomes of 9·5 (95%CI: 4·1-14·7) per 1000 women. Increase in costs of €203 (95%CI: -552 to 881) per woman. Total average costs were €15872 compared to €11840 per woman.	More effective and more costly than comparator (may not be statistically significant)	“Treat all” (strategy 1) is more effective and more costly than no treatment (strategy 7). The difference in perinatal mortality and costs between these two options may not be statistically significant.
van Baaren 2018 ^59^	Tocolysis and steroids with tertiary centre transfer (“treat all” reference strategy) Comparator: No treatment (“treat none”)	Reduction in perinatal mortality (16·9 vs 18·8 deaths per 1000 women) and poor outcomes (91·8 vs 120·3 per 1000 women). Increase in average costs (€30,187 vs €24,952 per woman)	More effective and more costly than comparator	“Treat all” (strategy 1) is more effective and more costly than no treatment (strategy 7). Confidence intervals are not reported for the comparison between these two studies, so statistical significance cannot be determined.

a This table presents treatment options in each study that are relevant to this review (e.g. “treat all”, “treat none”, and “ACS only”). Categorisation considers the options presented here, and does not compare with other treatment options analysed in primary studies that are not relevant to this review objective.

## Discussion

This is the first systematic review examining the cost-effectiveness of ACS and tocolytics in the context of preterm birth management, either alone or in combination. We identified 35 studies, mostly conducted in high-income countries. Studies were of varying methodological quality, and used diverse study designs and methodological approaches. Those pertaining to tocolytics considered a variety of agents, some of which are not in widespread use in contemporary obstetric practice. Studies generally used short-term time horizons, and thus may not accurately reflect longer term health effects or consider all aspects of cost-effectiveness.

Available evidence suggests that ACS is probably cost-saving or cost-effective when administered to women at imminent risk of preterm birth prior to 34 weeks’ gestation, though the magnitude of its economic effects probably varies between settings. The 2015 WHO recommends ACS (dexamethasone or betamethasone) for women at risk of imminent preterm birth between 24 to 34 weeks’ gestation, provided that certain treatment criteria are met.^5^ The current review corroborates this recommendation, as the intervention is likely to be cost-effective in this gestational age range. While Simpson and Lynch initially hypothesised that ACS may increase hospitalisation costs by increasing newborn survival, their own study refuted this.^28^ Conversely, WHO does not recommend ACS for late preterm birth as there is still uncertainties about the balance between risks and benefits,^5^ though some high-resource countries have moved in favour of its use on the basis of the 2016 ALPS trial.^63^, ^64^, ^65^ We found conflicting evidence from the USA as to whether this practice is likely to be cost-effective. Conclusions varied from ACS being dominant, cost-effective or dominated compared to no ACS, depending whether a full course was administered, and which newborn health outcomes were evaluated.^32^^,^^34^^,^^66^ The conflicting results reported by two studies using the same trial data illustrates the impact of study design and scope on cost-effectiveness outcomes.^32^^,^^34^

Given the methodological diversity of cost-effectiveness studies involving tocolytics, it was not possible to identify the best option(s) from an economic perspective. There was no clear consensus as to which tocolytic is economically superior when used to delay birth by at least 48 hours to facilitate ACS administration. Notably, older studies considered tocolytic options such as injectable terbutaline and magnesium sulfate; terbutaline has since been given a black box warning by the Food and Drug Administration,^67^ and a 2014 Cochrane review suggests magnesium sulfate is not an effective tocolytic agent.^15^ Studies in the current review suggest that when subcutaneous terbutaline is used for maintenance tocolysis, it not only prolongs pregnancy but decreases neonatal morbidity and costs when compared to oral tocolytics or placebo; however, these studies were of low methodological quality, several used the same data source, and maternal side effects were not considered.^46^, ^47^, ^48^, ^49^, ^50^ In addition, the efficacy of maintenance tocolysis in terms of health benefits to the neonate is itself uncertain.^5^^,^^68^

ACS and tocolytics are often used in combination in clinical care, and several studies considered the cost-effectiveness of this combination. While available studies indicated that women treated with both interventions generally had better health outcomes than no treatment, studies disagreed as to whether the combination of the two treatments saved or added costs; ACS and tocolytics in combination may nevertheless be cost-effective depending on decision-makers’ willingness-to-pay.

Strengths of this systematic review include the use of a broad search strategy across multiple databases, augmented by additional reference checks. We adhered to PRISMA guidance in terms of duplicate screening, data extraction and quality assessment – the latter conducted using the CHEERS checklist recommended by Cochrane.^23^ A limitation of this review is the inherent difficulty of comparing cost-effectiveness studies which differ greatly in terms of model composition, data sources, time horizons, outcomes examined, currency, and year of costs, as well as reflecting a diversity of health systems and payment arrangements.^69^ Notably, studies used different definitions of preterm labour and newborn health outcomes, limiting the opportunity to synthesise findings. We could not calculate a statistical measure of this heterogeneity as we did not produce pooled estimates, however we assume that heterogeneity is high given the differences between included studies in participants, interventions, outcomes and study design.

A number of included studies were quite old (9 studies were published prior to 2000), hence caution should be taken in generalizing these findings to contemporary health services, considering that treatment options, clinical decision-making, costs and payer arrangements can change over time. However, it was notable that studies that assessed ACS prior to 34 weeks’ gestation both before and after 2000 concluded that it was dominant. For tocolytics, we identified no studies of nifedipine or atosiban prior to 2000. While studies may conclude an intervention is cost-effective or cost-saving, this may not generalise to other settings (especially limited-resource settings) with different payer arrangements, higher costs of labour, hospital admission, supplies or equipment, or settings with more contemporary healthcare services. Some ACS and tocolytic options – such as betamethasone and atosiban – are not routinely available or used in many countries.

Further, robust cost-effectiveness studies are needed for these critical interventions in the context of preterm birth management. This review indicates ACS prior to 34 weeks’ gestation appears to be cost-effective, which can inform the decision-making of policymakers and maternal health program administrators on resource allocation, particularly in high income countries. Additional confirmatory evidence – particularly for limited-resource settings, where the burden of preterm-associated newborn mortality is often greater – would be useful to support ACS implementation and scale-up activities. Regarding ACS use between 34 and <37 weeks’ gestation, the conflicting economic evidence reflects the underlying uncertainty regarding health benefit (reduced respiratory morbidity) and harm (neonatal hypoglycaemia) trade-offs. The ALPS trial was conducted in tertiary care hospitals in the USA and it is not yet clear if the findings are applicable to lower-resource settings.^70^ If the health benefit-harm profile is more clearly established through additional trials, future cost-effectiveness analyses will be better positioned to fully evaluate the economic implications. In addition, observational studies have recently reported longer-term harms associated with ACS use, particularly when ACS-exposed babies are born at term or near-term,^71^^,^^72^ highlighting the importance of considering longer-term outcomes in future cost-benefit analyses. Such analyses would ideally explore how ACS cost-effectiveness might vary for different weeks of gestation.

The 2015 WHO guidelines indicate that if tocolytics are used, oral nifedipine is the preferred first-line option;^5^ however, on the basis of available evidence we were not able to determine if nifedipine was more cost-effective than other tocolytics. Future economic evaluations should consider the cost-effectiveness of tocolytics that have been shown to have superior clinical effects (such as nifedipine or atosiban). Such analyses could also consider the cost-effectiveness of these specific tocolytics and ACS alone or in combination. This systematic review was conducted in the context of updating WHO's 2015 recommendations on ACS and tocolytics for preterm birth,^73^ and will thus support WHO guideline developers and panels to make evidence-informed judgements on resource use and cost-effectiveness.

Available cost-effectiveness studies suggest ACS prior to 34 weeks’ gestation in women at risk of imminent preterm birth are probably cost-effective, while findings on the cost-effectiveness of ACS at 34 to <37 weeks’ gestation are contradictory depending on which newborn health outcomes are considered. While there are diverse cost-effectiveness studies for different types and indications for tocolysis, the available evidence is insufficient to conclude which tocolytic is superior in terms of cost-effectiveness. Further studies are needed, particularly for tocolytics alone and ACS and tocolytics in combination.

## Contributors

JPV, KEE and ES formulated the research question and developed the protocol, which was revised by NS, DC and OTO. ES, CB, STC, RIZ, JPV contributed to screening. ES, AE, CB and RIZ contributed to data extraction and quality assessment. NS and KE assisted with data analysis. ES, CB, AE, KEE, STC, RIZ, NS, DC, OTO, JPV all reviewed and commented on preliminary and final analysis findings. The manuscript was initially drafted by ES, KEE and JPV, and subsequently revised by CB, AE, KEE, STC, RIZ, NS, DC and OTO. All authors approved the final version of the manuscript.

## Declaration of interests

This work was supported by a grant to the Burnet Institute (where ES, CB, AE, KEE, STC, NS, JPV are affiliated) from UNDP/UNFPA/UNICEF/WHO/World Bank Special Programme of Research, Development and Research Training in Human Reproduction (HRP), a co-sponsored program of the World Health Organization (where DC and FO are employees). The authors declare that they have no competing interests.

## Data sharing statement

All data extracted from studies identified in this review are available in the Supplementary Appendix.

## Appendix Supplementary materials

Image, application 1
