# Supplementary material for: Cost-effectiveness of antenatal corticosteroids and tocolytic agents in the management of preterm birth: A systematic review
Source: eClinicalMedicine. 2022 Jun 3;49:101496. doi: 10.1016/j.eclinm.2022.101496 (PMC9167884; doi:10.1016/j.eclinm.2022.101496)
Supplement: Supplementary file 1 [file mmc1.docx]

# Research in context

## Evidence before this study

Efficacy evidence indicates that antenatal corticosteroids (ACS) prior to 34 weeks’ gestation for women at risk of imminent preterm birth significantly reduces neonatal morbidity and mortality. Though there is relatively less evidence on effects of ACS in the late preterm period (34 to <37 weeks’ gestation), they might reduce neonatal respiratory morbidity but could also increase neonatal hypoglycaemia. Multiple drug classes have been evaluated for tocolysis in women with spontaneous preterm labour. Some tocolytic drugs can effectively prolong pregnancy – providing time for ACS administration and/or transfer to higher level care – but tocolytic drugs have not yet been shown to independently improve substantive perinatal health outcomes. We identified a 2009 health technology assessment that broadly evaluated the economic effects of test-treatment interventions in preterm labour, however the cost-effectiveness of ACS and/or tocolytics only were not specifically reported.

## Added value of this study

We searched MEDLINE, Embase and a repository of maternal health economic evaluations derived from six economic and health databases. Available economic studies of ACS and/or tocolytics were largely conducted in high-income countries. ACS prior to 34 weeks’ gestation appears cost-effective, though economic evidence from the USA on ACS use in late preterm birth indicates that its cost-effectiveness varies depending on which health outcomes are considered. Some studies suggest that tocolysis to facilitate ACS administration was not cost-saving, but may be cost-effective. No single tocolytic option was identified as dominant in the management of spontaneous preterm labour.

## Implications of all the available evidence

ACS prior to 34 weeks’ gestation is cost-effective in high-income countries. There is limited economic evidence from low-to-middle-income countries, though modelling suggests ACS implementation and scale up would likely be cost-effective in these contexts. In light of the limited and conflicting evidence on tocolytics for spontaneous preterm labour, it is not possible to conclude what (if any) tocolytic option is the most cost-effective. Further, robust economic evaluations on ACS at 34-<37 weeks’ gestation, tocolytics alone, and ACS and tocolytics in combination are required, particularly those that explore cost-effectiveness in resource-limited settings.
